# The Interaction between* GSTT1*,* GSTM1*, and* GSTP1* Ile105Val Gene Polymorphisms and Environmental Risk Factors in Premalignant Gastric Lesions Risk

**DOI:** 10.1155/2017/7365080

**Published:** 2017-01-15

**Authors:** Anca Negovan, Mihaela Iancu, Valeriu Moldovan, Simona Mocan, Claudia Banescu

**Affiliations:** ^1^University of Medicine and Pharmacy, Tirgu Mureș, Gheorghe Marinescu 38, 540139 Mures, Romania; ^2^University of Medicine and Pharmacy “Iuliu Hațieganu” Cluj-Napoca, 8 Victor Babeş, 400012 Cluj-Napoca, Romania; ^3^Emergency County Hospital, Tirgu Mureș, Gheorghe Marinescu 50, 540136 Mures, Romania; ^4^Center for Advanced Medical and Pharmaceutical Research, University of Medicine and Pharmacy, Tirgu Mureș, Gheorghe Marinescu 38, 540139 Mures, Romania

## Abstract

The study investigated the possible influence of* GSTM1*,* GSTT1*, and* GSTP1* gene polymorphisms as predisposing factors for premalignant gastric lesions as well as their interaction with* H. pylori* infection, gastrotoxic drugs, smoking, and alcohol consumption. In this study, 270 patients with a complet set of gastric biopsies and successfully genotyped were finally included. The GSTM1 gene polymorphism had significant contribution in mild/severe endoscopic lesions (*p* = 0.01) as well as in premalignant lesions (*p* = 0.01). The* GSTM1* null genotype increased the risk for mucosal defects in* H. pylori*-negative patients (OR = 2.27, 95% CI: 1.20–4.37) and the risk for premalignant lesions in patients with no alcohol consumption (OR = 2.13, 95% CI: 1.19–3.83). The* GSTT1* deleted polymorphism did not significantly increase the risk for premalignant lesions in the absence of gastrotoxic drugs (OR = 1.82, 95% CI: 0.72–4.74). The combined* GSTT1T1* and* GSTM1* null polymorphisms were borderline correlated with an increased risk for premalignant lesions (OR = 1.72, 95% CI: 1.00–2.97). The wild-type* GSTP1* Ile/Ile genotype versus the variant genotypes Ile/Val + Val/Val was significantly associated with a decreased risk of gastric atrophy/intestinal metaplasia (OR = 0.60, 95% CI: 0.37–0.98). In conclusion, the* GSTM1* and* GSTT1* null genotypes increased the risk for premalignant and endoscopic gastric lesions, modulated by* H. pylori*, alcohol, or gastrotoxic drug consumption, while the presence of the GSTP1Val allele seemed to reduce the risk for premalignant lesions.

## 1. Introduction

It is widely accepted today that gastric carcinogenesis is a multistep and multifactorial process, influenced by interactions between the host's genetic susceptibility and environmental factors. For the intestinal type of gastric cancer the role of* Helicobacter pylori (H. pylori)* infection and histopathology of the precancerous lesions (chronic gastritis, gastric atrophy (GA), intestinal metaplasia (IM), and epithelial dysplasia (ED)) have long been accepted [1, 2]. An important role seems to play the interaction between* H. pylori* (and its virulence) infection as a triggering factor and the host's genetic susceptibility [3]. Although numerous studies have investigated genetic polymorphisms in gastric cancer, little has been performed related to precancerous gastric lesions.

Glutathione S-transferases (GSTs) are the most important enzymes of the phase II metabolizing xenobiotic pathway, which detoxifies several cytotoxic compounds [4]. They are involved in the metabolism of carcinogens, drugs, and reactive oxygen species (ROS) playing a protective role against the oxidative damage of DNA [5]. They have been grouped into at least seven classes called *α* (alpha), *μ* (mu), *π* (pi), *σ* (sigma), *ω* (omega), *θ* (theta), and *ζ* (zeta) [6–8]. Glutathione S-transferase T1 (*GSTT1*) and glutathione S-transferase M1 (*GSTM1*) are members of the *θ* and *μ* classes, respectively, and have been shown to be polymorphic. The common variant of* GSTM1* and* GSTT1* genes is the homozygous deletion (null genotype) which leads to reduced enzyme activity and increased risk for various diseases, including esophageal, gastric, or colon cancer [9, 10]. For glutathione S-transferase P1 (GSTP1) the single-nucleotide polymorphisms (SNPs) in the* GSTP1* gene, resulting in amino acid substitutions at codons 105 (Ile→Val) and 114 (Val→Ala), have been associated with diminished GST enzyme activity [8, 9, 11]. In numerous studies the* GSTM1*,* GSTT1*, and* GSTP1* gene polymorphisms have been investigated for their possible role in risk occurrence of various diseases, including gastric cancer [5]. The roles of variant GST polymorphisms were questioned especially in interaction with environmental recognized risk factors for gastric cancer (*H. pylori* infection, smoking, alcohol consumption, or salt intake) [12–16]. At present, the studies' results are inconclusive and there are no defined genetic markers having important roles in the progression thorough the carcinogenesis cascade [3, 12–18].

The objectives of our study were (i) to investigate the influence of* GSTM1*,* GSTT1*, and* GSTP1* Ile105Val gene polymorphisms on the risk of gastric precancerous lesions and (ii) to test the possible interaction effect between genetic and environmental factors (*H. pylori* current infection, smoking, alcohol, and drug consumption) in histologic and endoscopic gastric lesions in Romanian population.

## 2. Materials and Method

### 2.1. Subjects

Consecutive patients referred for upper digestive endoscopies (UDE) to the 3rd Medical Clinic of the Tirgu Mures Emergency County Hospital, Romania, were screened for study inclusion. Patients were examined for dyspeptic symptoms, anemia, or screened for gastrointestinal bleeding risk (before major cardiovascular surgery or before the start of antithrombotic therapies). Clinical and demographical data were collected by structured interview, clinical examination, and reviewing of medical records. We considered, as drinkers, patients consuming at least 10 units (10 mL) of pure alcohol weekly and nondrinkers patients consuming any amount of alcohol below this level. Patients smoking more than 5 cigarettes/day including quitters during the past 5 years were considered as smokers. We recorded the digestive symptoms, namely, upper abdominal pain, heartburn, regurgitation, nausea, vomiting, and early satiety. We considered gastrotoxic drug exposure the nonsteroidal anti-inflammatory drugs (NSAIDs) consumption as regular daily doses in patients with arthritis or other inflammatory disorders who needed chronic therapy (more than six months). Long-term antiplatelet therapy (low-dose aspirin 75–325 mg/day; clopidogrel 75 mg/day more than 6 months) was also considered gastrotoxic medication. We excluded patients with acute bleeding episodes, previous therapy for eradication of* H. pylori* infection, gastric surgery, gastric or esophageal cancer, and esophageal varices, patients with severe medical conditions (cancer, cirrhosis, severe heart or renal failure, etc.), and patients with lacking data (biopsies, social habits, and drug exposure).

The Ethical Committee of the University of Medicine and Pharmacy of Tirgu Mures, Romania, approved the study and a written informed consent was obtained from all subjects included.

### 2.2. Genotyping

Blood samples were used for rapid extraction of genomic DNA.* GSTM1* and* GSTT1* gene polymorphisms were analyzed by the use of multiplex polymerase chain reaction as described previously [19]. The* GSTP1* Ile105Val gene polymorphism was investigated by PCR-RFLP (polymerase chain reaction and restriction fragment length polymorphism) method as previously described [20].

Genotyping was successfully performed in 373 cases. Genotype frequencies of* GSTP1* gene polymorphisms did not deviate significantly from the expected frequencies of Hardy-Weinberg equilibrium on each subgroup (*p* = 0.23 in group without endoscopic lesions, resp., *p* = 0.964 in group without premalignant lesions).

Finally, there were 270 patients included in the analysis; controls were frequency-matched with the cases by age ±5 years.

### 2.3. Endoscopy

Endoscopy was carried out in every patient by an endoscopist blinded to drug exposure and symptoms. We described mucosal lesions as erythema, petechiae, erosions, or ulcers. Petechiae were defined as hemorrhagic areas with no mucosal defect and erosions as mucosal defects smaller than 5 mm. Defects larger than 5 mm in diameter, extended into the deeper layers of the gastric or duodenal wall, were defined as ulcer. Endoscopic mucosal lesions were classified as mild if only erythema, petechiae, or less than two erosions were observed on endoscopic examination. We considered severe endoscopic lesions the presence of more than three erosions or ulcer in the gastroduodenal mucosa.

Two biopsy specimens from the antrum and two from the corpus (from the lesser and the greater curvature) were obtained for routine histology in every patient. Two pathologists also blinded to patient drug exposure and symptoms examined them.

### 2.4. Histology

Biopsy specimens were fixed in 10% buffered formalin, routinely processed, embedded in paraffin, and stained with hematoxylin-eosin, PAS-alcian blue, and Giemsa.* H. pylori* infection was considered negative if* H. pylori* were absent from all biopsy sites and positive if at least one histology test was positive. The degrees of mucosal chronic inflammation, activity,* H. pylori* infection, glandular atrophy, and intestinal metaplasia were classified into 4 grades according to the Updated Sydney System. It has long been recognized that intestinal metaplasia (IM) is heterogeneous, and several classifications have been proposed. We considered complete metaplasia when the epithelium resembles the small intestinal phenotype with eosinophilic enterocytes displaying a well-defined brush border and well-formed goblet cells and incomplete metaplasia the presence of a colonic epithelium phenotype with multiple, irregular mucin droplets of variable size in the cytoplasm and absence of a brush border. We also evaluated dysplasia according to the modified Vienna classification, but patients with dysplasia or neoplasia were excluded. Patients without important inflammation, but with prominent foveolar hyperplasia, fibromuscular replacement of the lamina propria, and congestion of superficial mucosal capillaries, were diagnosed as reactive gastropathy. We did not include patients with autoimmune gastritis or with an incomplete set of biopsies.

### 2.5. Statistical Analysis

The quantitative variables representing demographic characteristics were expressed as mean ± standard deviation while studied environmental and gene polymorphisms factors were summarized by absolute and relative frequencies. The differences in distribution of demographic variables and selected gene polymorphisms between cases (mild or sever endoscopic lesions and gastric atrophy or intestinal metaplasia) and controls (without endoscopic lesions and without gastric atrophy) were tested by Student's *t*-test and Chi-square test.

The associations between studied gene polymorphisms and the risk of gastric lesions were tested by logistic regression analysis. The magnitude of association was quantified by the multivariable odds ratio and their 95% confidence intervals. We evaluated the gene-environment interaction using a multivariable multiplicative model composed by age, sex, environmental, genetic factors, and the interaction term of interest.

The level of statistical significance for all two-sided tests was set to 0.05.

The advanced environment for statistical computing R (v.3.3.1, Vienna, Austria) was used for statistical analysis.

## 3. Results

### 3.1. Description of Sample Selected Characteristics

The study included 270 patients successfully genotyped with a complete set of data. The distribution of demographic and clinical characteristics in the studied group is described in Table 1. The repartition of age values and sex frequency was homogenous in all studied subgroups (*p* > 0.05). There was no significant difference in the distribution of* H. pylori* and alcohol consumption between patients without endoscopic lesions and patients with mild or severe endoscopic lesions (*p* > 0.05). There was no significant association between these factors and gastric atrophy or intestinal metaplasia (*p* > 0.05). Antiplatelet or NSAIDs consumption was significantly associated with mild or severe endoscopic lesions (*p* = 0.011). Premalignant lesions were associated with smoking habits (*p* = 0.002), but not with* H. pylori* active infection or alcohol consumption.

### 3.2. Association between Selected Polymorphisms and Risk of Endoscopic Lesions or Premalignant Gastric Lesions (Gastric Atrophy or Intestinal Metaplasia)

There was no significant difference regarding the frequency distribution of* GSTP1* Ile105Val,* GSTT1*, and* GSTM1* variant genotypes in patients without endoscopic lesions versus patients with mild or severe endoscopic lesions (49.7 versus 39.6%, *χ* ^2^(1) = 2.60, *p* = 0.130 for* GSTP1*  Ile105Val; 20.7% versus 20.8%, *p* = 0.987 for GSTT1; and 46.7% versus 56.4%, *χ* ^2^(1) = 2.60, *p* = 0.130 for GSTM1).

The variant genotypes Ile/Val + Val/Val of* GSTP1* Ile105Val were borderline associated with less frequent premalignant lesions than the wild-type Ile/Ile genotype (39.5% versus 51.8%, *χ* ^2^(1) = 4.06, *p* = 0.051). Frequency distributions for the* GSTT1* and* GSTM1* null genotypes were comparable in patients with gastric atrophy/intestinal metaplasia compared with patients without premalignant lesions (19.4% versus 22.0%, *χ* ^2^(1) = 0.28, *p* = 0.653; 55.8% versus 45.4%, *χ* ^2^(1) = 2.93, *p* = 0.090, resp.).

The concomitant presence of* GSTT1* and* GSTM1* null genotype was similar in patients with mild or severe endoscopic lesions versus patients without lesions (9.9% versus 8.9%, *χ* ^2^(3) = 2.54, *p* = 0.471). Analogue results were obtained for patients with gastric atrophy/intestinal metaplasia compared with patients without premalignant lesions (8.5% versus 9.9%, *χ* ^2^(3) = 4.02, *p* = 0.260).

Compared with the wild-type Ile/Ile genotype, the variant Ile/Val + Val/Val genotypes of* GSTP1* Ile105Val gene polymorphism were significantly associated with a decreased risk of gastric atrophy/intestinal metaplasia (adjusted OR = 0.60, 95% CI: [0.37, 0.98]) after adjusting for age and sex. We also noticed an increased risk of gastric atrophy/intestinal metaplasia for* GSTM1* null genotype (adjusted OR = 1.55, 95% CI: [0.96, 2.53]) with a tendency towards statistical significance *p* = 0.074 (Table 2). Presence of the double null genotypes of* GSTM1* and* GSTT1* was borderline associated with an increased risk of premalignant lesions (*p* = 0.05, adjusted OR = 1.72, 95% CI: [1.00, 2.97]).

### 3.3. Interaction Effect between Environmental Factors and the Selected Gene Polymorphisms on Gastric Lesions

As shown in Table 3, there was a significant interaction between the* GSTM1* polymorphism and* H. pylori* (*p* = 0.038). The logistic regression results (the estimation of regression coefficients is not presented) calculated a significant contribution of the* GSTM1* gene polymorphism on mild or severe endoscopic lesions (*p* = 0.013) and the impact of the null gene polymorphism was different depending on* H. pylori* status (OR for interaction term = 0.31, 95% CI: [0.10, 0.93]). The* GSTM1* null genotype was associated with an increased risk for mild or severe endoscopic lesions in patients without* H. pylori* (OR = 2.27, 95% CI: [1.20, 4.37]) while in patients with* H. pylori* infection a decreased risk was observed, without statistical significance (OR = 0.70, 95% CI: [0.29, 1.67]). The logistic regression results also showed a significant contribution of the* GSTM1* gene polymorphism on the risk of premalignant lesions (*p* = 0.011) and the impact of the null gene polymorphism was different depending on alcohol consumption status (OR for interaction term = 0.27, 95% CI: [0.08, 0.94]). We noticed that the presence of the null genotype was associated with an increased risk odds ratio (OR = 2.13, 95% CI: [1.19, 3.83]) in patients without alcohol consumption, while in patients with alcohol consumption a decreased risk was observed, without statistical significance (OR = 0.58, 95% CI: [0.22, 1.54]).

The effect of the* GSTT1* deleted polymorphism on premalignant lesions was different depending on drug consumption status (OR for interaction term = 0.26, 95% CI: 0.07, 0.94). The* GSTT1* null genotype was associated with an increased risk, without statistical significance (OR = 1.82, 95% CI: 0.72, 4.74) in patients without drug intake, while* GSTT1* null genotype was associated with a decreased risk with no statistical significance (OR = 0.47, 95% CI: 0.21, 1.07) in patients with gastrotoxic treatments.

The plausible interaction effects justified the estimation of adjusted multivariable OR (Table 3) for the highlighted the magnitude of association.

## 4. Discussions

The most commonly deleted polymorphisms in the* GSTT1* and* GSTM1* genes associated with decreased detoxifying activity of the GST enzyme [4, 9] were extensively studied for gastric cancer occurrence and less frequently for premalignant lesions. On the other hand, the SNP in* GSTP1* gene resulting in amino acid substitutions at codon 105 (Ile→Val) is also associated with reduced detoxifying activity of the GST enzyme and cancer risk but less studied in premalignant gastric lesions. Many studies showed that the polymorphic variants of* GSTT1*,* GSTM1*, and* GSTP1* genes were associated with increased risk for gastric cancer, especially in the Asian population [13, 21–23]. However, certain studies performed in the European population sustained that the* GST* gene polymorphisms are not relevant in gastric cancer [15]. There is a paucity of information regarding the frequency and role of the mentioned polymorphism in gastric diseases in the Romanian population, characterized by a high prevalence of* H. pylori* infection and a high mortality related to gastric cancer [24], like in some Asian populations, but with a genetic European background.

The majority of previous studies sustained a decreased GST enzyme activity in the presence of* H. pylori* infection [25, 26]. As the variant genotype of* GSTP1* Ile105Val or null* GSTM1* or* GSTT1* genotypes were also reported to decrease the activity of the GST enzyme [8–11, 16], we investigated the possible interplay between GST gene polymorphisms and* H. pylori* infection in endoscopic and histologic gastric lesions. We questioned also the interplay between* GST* gene polymorphisms and the rest of environmental risk factors known to increase the susceptibility for endoscopic/histologic gastric lesions (gastrotoxic medication, smoking, and alcohol consumption).

The frequency of* GSTM1* null genotype was reported to range between 40 and 60% in the European population, as in our study (49.6%), while* GSTT1* null genotype was reported between 13–26% [15, 27], similar to our results (20.7%). There are wide ethnic differences in the frequency of* GSTP1* Ile105Val polymorphism, ranging from a frequency between 38 and 60% of* GSTP1* Ile/Val or Val/Val genotype in the European population to 15.2–61.5% in the Asian population [19, 28] and 45.9% in our study. The prevalence of premalignant gastric lesions in our studied population was 47.7%, intermediate in comparison with worldwide reported data, correlated with the regional prevalence of* H. pylori* infection, germ virulence, or host characteristics, but very high for the European region [29, 30].

The* GSTM1* null genotype tended to increase the risk for gastric atrophy/intestinal metaplasia, but this influence was surprisingly modified by alcohol consumption in our studied population. On the other hand, the* GSTM1* null genotype in the* H. pylori*-negative patients was associated with an increased risk for severe endoscopic lesions. Our surprising findings suggest a more complex gene-environment interaction in our population, proposing an independent role of the* GSTM1* deleted polymorphism for non-*H. pylori* endoscopic gastric lesions and premalignant gastric lesions. Our results are not similar with Chinese studies that failed to sustain role of* GSTM1* null genotype in premalignant gastric lesions, even in association with environmental factors [31]. Current observations sustain the possible role of* GSTM1* polymorphism in gastric diseases in our population, as a recent meta-analysis also proved its role in carcinogenesis in Caucasian population modulated by* H. pylori* infection and smoking [32].

The* GSTT1* null genotype seems to have no influence on endoscopic or premalignant gastric lesions, similar with other results in this respect [31]. Hence, in the presence of gastrotoxic drug consumption (NSAIDs and/or antiplatelet drugs) the presence of* GSTT1* null genotype decreased the risk for gastric atrophy/intestinal metaplasia. The mechanisms of this association are worth to be investigated as NSAIDs and aspirin was proven to protect against the risk of gastric cancer by a different cyclooxygenase 2 mediated pathway [33–35] in order to determine genetic factors that can be used to identified candidates for preventive therapy. The combined* GSTT1* T1 and* GSTM1* null genotypes were correlated with an increased risk for premalignant lesions, sustaining the greater influence of the* GSTM1* deleted polymorphism in our population, and the unusual effect of the* GSTT1* polymorphism in gastric lesions.

The variant genotypes Ile/Val or Val/Val of* GSTP1* Ile105Val in our patients were surprisingly correlated with less frequent premalignant lesions. Despite the proved role of the variant allele Val of* GSTP1* Ile105Val SNP in gastric cancer risk in some Asian populations, its role for the gastric premalignant lesions risk has been sustained only in subgroups >60 years or in association with* H. pylori* infection, smoking, or alcohol consumption in a Chinese study [36]. Despite the similar frequency of gastric atrophy in patients with variant* GSTP1* Ile105Val genotype in Chinese and Romanian populations (37% versus 35.7%) [34], environmental factors did not influence the role of this gene polymorphism in premalignant gastric lesions in our study. Our results need further investigation, as numerous complex gene-environment interactions in various diseases implying the* GSTP1* Ile105Val gene polymorphism were observed, with no clear mechanism [37].

The* GST* polymorphisms seem to play a role in gastric cancer disease in our population, modulated by exposure to various exogenous factors (smoking, alcohol, gastrotoxic drugs, and* H. pylori*), but probably with more complex gene-gene and gene-environment interactions than those already studied. Our results can be explained by different exposures to lifestyle risk factors and the different genetic background in various populations.

The limitations of our study were firstly the low number of cases in some subgroups used for stratified analysis, which led to relatively large 95% confidence intervals or lack of statistical significance in some cases. Secondly, we could not apply the multiplicative model for test interaction between combined genotype and environmental factors due to the low frequency of some specific classes in combined genotype* GSTM1* and* GSTT1*. Thirdly, for* H. pylori* infection we used only the histologic diagnosis, which can miss certain cases with less extensive germ colonization.

The strength of our study is the prospective evaluation of the proposed genetic host factors playing a role in gastric carcinogenesis in a specific ethnic population, in association with the most important clinical and pathological aspects. To the best of our knowledge this is the first study questioning the interplay between* GSTM1*,* GSTT1*, and* GSTP1* Ile105Val gene polymorphisms and environmental factors in gastric lesions in a Caucasian population.

In conclusion, the* GSTM1* and* GSTT1* null genotypes increased the risk for premalignant and endoscopic lesions in our population, modulated by* H. pylori*, alcohol, or gastrotoxic drug consumption, while presence of the* GSTP1*Val allele seemed to reduce the risk for gastric premalignant lesions.

## Figures and Tables

**Table 1:** The distribution of *H. pylori* infection, gastrotoxic medication, alcohol consumption, and smoking in the studied group.

	Without endoscopic lesions (*n* = 169)	Mild or severe endoscopic lesions (*n* = 101)	*p* value^*∗*^	Without premalignant lesions (*n* = 141)	With premalignant lesions (*n* = 129)	*p* value^*∗*^
Age (mean ± SD)	65.64 ± 9.60	66.12 ± 7.88	0.656	65.06 ± 8.07	66.65 ± 9.85	0.149
*≤60*	45	23	0.480	38	30	0.485
*>60*	124	78		103	99	
Sex						
*Female*	95	44	0.059	73	66	0.920
*Male*	74	57		68	63	
*H. pylori* infection						
*Negative*	119	66	0.386	99	86	0.531
*Positive*	50	35		42	43	
Gastrotoxic drugs^a^						
*No*	89	37	**0.011**	64	62	0.660
*Yes*	80	64		77	67	
Smoking^b^						
*Nonsmoker*	157	90	0.280	136	111	**0.002**
*Smoker*	12	11		5	18	
Alcohol^c^						
*No*	129	74	0.573	107	96	0.780
*Yes*	40	27		34	33	

SD = standard deviation; ^a^presence of NSAIDs or antiplatelet therapy; ^b^>5 cigarettes/day including quitters during the past 5 years; ^c^consumption of >10 units/week, ^*∗*^obtained from Student's *t*-test for independent samples or Chi-square test.

**Table 2:** Effect of the studied gene polymorphisms on the outcome variable.

	Without endoscopic lesions versus mild or severe endoscopic lesions (169/101)		*p* value^*∗*^	Adjusted OR^†^ [95% CI]	Without premalignant lesions versus with premalignant lesions (141/129)		*p* value^*∗*^	Adjusted OR^†^ (95% CI)
*GSTP1* Ile105Val								
Ile/Ile^a^	85	61			68	78		
Ile/Val + Val/Val	84	40	0.116	0.67 [0.4, 1.11]	73	51	**0.038**	**0.60 [0.37, 0.98]**
*GSTT1*								
T1^a^	134	80			110	104		
Null	35	21	0.844	0.94 [0.50, 1.74]	31	25	0.627	0.86 [0.47, 1.57]
*GSTM1*								
M1^a^	90	44			77	57		
Null	79	57	0.106	1.51 [0.92, 2.52]	64	72	**0.074**	1.55 [0.96, 2.53]
*GSTT1/M1*								
T1/M1	70	33			60	43		
T1/null	64	47	0.115	1.58 [0.90, 2.79]	50	61	**0.050**	1.72 [1.00, 2.97]
Null/M1	20	11	0.873	1.07 [0.44, 2.49]	17	14	0.774	1.15 [0.50, 2.58]
Null/null	15	10	0.492	1.38 [0.54, 3.49]	14	11	0.757	1.15 [0.46, 2.84]

^a^Reference category; CI = confidence level: [lower limit; upper limit]; ^†^OR was adjusted by age and sex.

**Table 3:** The gene-environment interaction in endoscopic and premalignant gastric lesions.

Factors	Genotype	Without endoscopic lesions versus mild or severe endoscopic lesions	Adjusted OR^‡^ [95% CI]	Without premalignant lesions versus with premalignant lesions	Adjusted OR^‡^ (95% CI)
*H. pylori*	*GSTP1 *Ile105Val				
*Negative*	Ile/Ile	56/40	1 (reference)	44/52	1 (reference)
	Ile/Val + Val/Val	63/26	0.55 [0.29, 1.04]	55/34	0.47 [0.26, 0.87]
*Positive*	Ile/Ile	29/21	1.00 [0.48, 2.07]	24/26	0.86 [0.42, 1.77]
	Ile/Val + Val/Val	21/14	0.70 [0.32, 1.54]	18/17	0.56 [0.26, 1.22]
		*p* value for interaction = 0.676		*p* value for interaction = 0.568	
*H. pylori*	*GSTT1*				
*Negative*	T1	96/49	1 (reference)	77/68	1 (reference)
	Null	23/17	1.40 [0.65, 2.98]	22/18	1.00 [0.47, 2.12]
*Positive*	T1	38/31	1.39 [0.75, 2.57]	33/36	1.06 [0.58, 1.57]
	Null	12/4	0.53 [0.16, 1.73]	9/7	0.71 [0.25, 2.01]
		*p* value for interaction = 0.087		*p* value for interaction = 0.576	
*H. pylori*	*GSTM1*				
*Negative*	M1	69/26	1 (reference)	56.39	1 (reference)
	Null	50/40	2.23 [1.20, 4.37]	43/47	1.66 [0.90, 3.07]
*Positive*	M1	21/18	2.07 [0.92, 4.69]	21/18	1.04 [0.47, 2.30]
	Null	29/17	1.46 [0.69, 3.09]	21/25	1.57 [0.77, 3.19]
		*p* value for interaction = **0.038**		*p* value for interaction = 0.858	
Alcohol^a^	*GSTP1* Ile105Val				
*No*	Ile/Ile	67/47	1 (reference)	52/62	1 (reference)
	Ile/Val + Val/Val	62/27	0.60 [0.32, 1.11]	55/34	0.49 [0.27, 0.87]
*Yes*	Ile/Ile	18/14	0.78 [0.32, 1.90]	16/16	0.64 [0.26, 1.55]
	Ile/Val + Val/Val	22/13	0.44 [0.20, 0.96]	18/17	0.42 [0.20, 0.90]
		*p* value for interaction = 0.909		*p* value for interaction = 0.628	
Alcohol^a^	*GSTT1*				
*No*	T1	104/63	1 (reference)	22/14	1 (reference)
	Null	25/11	0.72 [0.31, 1.60]	85/82	0.72 [0.33, 1.54]
*Yes*	T1	30/17	0.59 [0.25, 1.35]	9/11	0.59 [0.25, 1.34]
	Null	10/10	1.01 [0.32, 2.07]	25/22	0.85 [0.34, 2.16]
		*p* value for interaction = 0.214		*p* value for interaction = 0.328	
Alcohol^a^	*GSTM1*				
*No*	M1	74/32	1 (reference)	64/42	1 (reference)
	Null	55/42	1.83 [1.00, 3.37]	43/54	2.13 [1.19, 3.83]
*Yes*	M1	16/12	1.08 [0.41, 2.76]	13/15	1.40 [0.54, 3.67]
	Null	24/15	0.99 [0.46, 2.13]	21/18	0.81 [0.28, 1.53]
		*p* value for interaction = 0.256		*p* value for interaction = **0.041**	
Smoking^b^	*GSTP1 *Ile105Val				
*No*	Ile/Ile	78/58	1 (reference)	66/70	1 (reference)
	Ile/Val + Val/Val	79/32	0.51 [0.29, 0.89]	70/41	0.51 [0.30, 0.87]
*Yes*	Ile/Ile	7/3	0.76 [0.15, 3.17]	2/8	5.14 [1.15, 36.52]
	Ile/Val + Val/Val	5/8	1.97 [0.61, 6.33]	3/10	3.46 [0.91, 13.13]
		*p* value for interaction = 0.090		*p* value for interaction = 0.790	
Smoking^b^	*GSTT1*				
*No*	T1	126/72	1 (reference)	107/91	1 (reference)
	Null	31/18	1.06 [0.53, 2.10]	29/20	0.94 [0.48, 1.82]
*Yes*	T1	8/8	2.23 [0.70, 7.14]	3/13	7.36 [2.04, 35.67]
	Null	4/3	1.21 [0.26, 5.56]	2/5	3.87 [0.73, 20.43]
		*p* value for interaction = 0.503		*p* value for interaction = 0.609	
Smoking^b^	*GSTM1*				
*No*	M1	87/40	1 (reference)	76/51	1 (reference)
	Null	70/50	1.68 [0.97, 2.93]	60/60	1.67 [0.99, 2.85]
*Yes*	M1	3/4	3.69 [0.71, 21.11]	1/6	11.08 [1.68, 220.49]
	Null	9/7	2.17 [0.75, 6.24]	4/12	7.96 [2.43, 26.06]
		*p* value for interaction = 0.294		*p* value for interaction = 0.509	
Drugs^c^	*GSTP1* Ile105Val				
*No*	Ile/Ile	46/25	1 (reference)	31/40	1 (reference)
	Ile/Val + Val/Val	43/12	0.46 [0.19, 1.05]	33/22	0.42 [0.19, 0.89]
*Yes*	Ile/Ile	39/36	1.67 [0.85, 3.34]	37/38	0.79 [0.40, 1.55]
	Ile/Val + Val/Val	41/28	1.18 [0.60, 2.34]	40/29	0.50 [0.26, 0.98]
		*p* value for interaction = 0.439		*p* value for interaction = 0.434	
Drugs^c^	*GSTT1*				
*No*	T1	74/28	1 (reference)	54/48	1 (reference)
	Null	15/9	1.65 [0.61, 4.26]	10/14	1.82 [0.72, 4.74]
*Yes*	T1	60/52	2.41 [1.33, 4.42]	56/56	1.23 [0.70, 2.18]
	Null	20/12	1.59 [0.69, 3.67]	21/11	0.58 [0.25, 1.33]
		*p* value for interaction = 0.163		*p* value for interaction = **0.042**	
Drugs^c^	*GSTM1*				
*No*	M1	48/19	1 (reference)	36/31	1 (reference)
	Null	41/89	1.23 [0.55, 2.74]	28/31	1.49 [0.71, 3.16]
*Yes*	M1	42/25	1.61 [0.76, 3.45]	41/26	0.88 [0.43, 1.80]
	Null	38/39	2.95 [1.47, 5.91]	36/41	1.51 [0.78, 2.91]
		*p* value for interaction = 0.457		*p* value for interaction = 0.782	

^a^>10 units/week; ^b^>5 cigarettes/day including during the past 5 years; ^c^presence of NSAIDs or antiplatelet therapy; ^‡^OR was adjusted by age, sex, *H. pylori*, smoking, alcohol, and NSAIDs or antiplatelet therapy.
